# “Pseudo-Spleen Sign” on Gastric Ultrasound in the Supine Position: A Case Report of a Patient With Delayed Gastric Emptying

**DOI:** 10.7759/cureus.103490

**Published:** 2026-02-12

**Authors:** Satoru Sekiya, Satoshi Jujo, Shosei Ro, Hiroshi Okamoto

**Affiliations:** 1 Department of Critical Care Medicine, St. Luke’s International Hospital, Tokyo, JPN; 2 Department of Anesthesiology, Kameda Medical Center, Kamogawa, JPN; 3 Department of Pulmonary Medicine, Thoracic Center, St. Luke’s International Hospital, Tokyo, JPN

**Keywords:** enteral nutrition (en), gastric point-of-care ultrasound, gastric ultrasound, nutrition in critical care, point-of-care ultrasound (pocus)

## Abstract

Gastric ultrasound (GUS) plays a key role in identifying delayed gastric emptying and preventing pulmonary aspiration. Conventional GUS estimates gastric volume by measuring the cross-sectional area (CSA) of the antrum in the right lateral decubitus (RLD) position, which is often impractical in respiratory-unstable patients. We report an adult patient in whom GUS in the supine position successfully identified a distended gastric fundus filled with enteral nutrition (EN). In this patient, GUS in the RLD position was not feasible due to a respiratory condition. However, a daily supine abdominal ultrasound using the left upper quadrant (LUQ) view revealed a distended gastric fundus with EN. This finding suggested delayed gastric emptying; thus, we reduced the continuous EN infusion rate. This adjustment, in combination with prokinetic agents and continued clinical monitoring, may have contributed to preventing reflux and aspiration. The ultrasound appearance of the fundus resembled a spleen, and we termed it “pseudo-spleen sign.” This supine GUS approach with this novel finding may offer a practical adjunct to conventional GUS that requires the RLD position.

## Introduction

Gastric ultrasound (GUS) plays a key role in identifying delayed gastric emptying and preventing pulmonary aspiration, especially in critically ill patients receiving enteral nutrition (EN) [1,2]. Conventional GUS estimates gastric volume by measuring the cross-sectional area (CSA) of the gastric antrum in the right lateral decubitus (RLD) position, using the following formula: gastric volume (mL) = 27 + (14.6 × RLD - CSA [cm²]) - (1.28 × age) [3,4]. According to the European Society for Clinical Nutrition and Metabolism (ESPEN) guideline and several studies, EN is considered delayed or suspended when gastric residual volume (GRV) is >500 mL/six hours or >200 mL/four hours [2,5]. However, the conventional GUS relies on antral measurements in the RLD position, which is often impractical in patients with respiratory compromise or requiring mechanical ventilation [6]. Therefore, an alternative or adjunctive GUS approach to visualize the stomach is warranted when the antral GUS is not feasible or inconclusive. GUS can assess three gastric locations: the antrum, body, and fundus [7]. Antral GUS in the RLD position provides a reliable quantitative assessment, whereas gastric fundus ultrasound (fundus GUS) is unreliable for quantitative evaluation [7]. However, a previous study suggested that fundus GUS performed in the supine position can provide qualitative information about gastric content volume [8]. In this case report, we performed fundus GUS in the supine position and successfully identified a distended gastric fundus filled with enteral nutrition in an adult patient.

## Case presentation

A man in his 60s with respiratory failure due to right empyema associated with right lung cancer was admitted to the intensive care unit (ICU) after unsuccessful surgical thoracic drainage because of extensive intrathoracic adhesions. On ICU admission, he was hemodynamically unstable and required vasopressor support and invasive mechanical ventilation for respiratory failure. His Sequential Organ Failure Assessment (SOFA) score was 9. He had no history of gastrointestinal surgery, alcohol misuse, or familial metabolic disorders. Sedation was maintained with continuous infusions of midazolam and fentanyl, and neuromuscular blockade was achieved using rocuronium. EN via a feeding tube was initially withheld because of ongoing vasopressor requirements.

After partial improvement in respiratory status and a reduction in vasopressor dose, we introduced continuous EN on postoperative day (POD) 9 at a rate of 10 mL/hour, while low-dose vasopressor support was still in place. On POD 10, we increased the EN rate to 20 mL/hour, but the aspirated GRV reached 220 mL over eight hours. In response, we administered prokinetic therapy. On POD 11, we increased the EN rate to 30 mL/hour, resulting in a further rise in GRV exceeding 350 mL over eight hours. We considered conventional GUS in the RLD position to estimate gastric content volume; however, because of concerns about respiratory deterioration, we instead performed GUS in the supine position (supine-based posture with approximately 30° head-up elevation) to measure the antral CSA. In the examination, the antrum appeared small, round, and non-distended, showing a typical bull’s-eye appearance. If we had obtained this finding in the RLD position, we would have concluded no delayed gastric emptying; however, because of the low diagnostic sensitivity of supine antral assessment, we were unable to exclude delayed gastric emptying [7].

**Figure 1:**
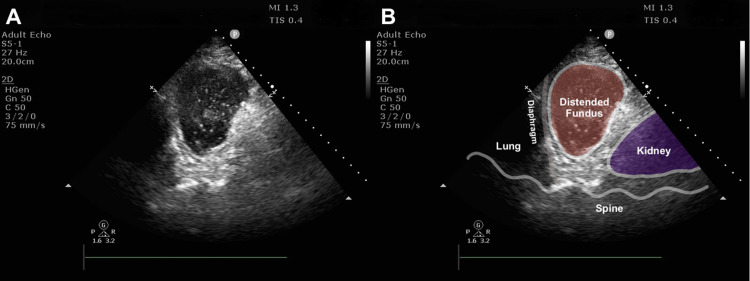
Distended gastric fundus filled with EN Ultrasound images of the gastric fundus obtained from the intercostal trans-splenic LUQ view in the supine position. (A) Gastric ultrasound image demonstrating a distended gastric fundus filled with EN. (B) The same ultrasound image with annotation illustrating the distended fundus and surrounding anatomical landmarks EN, enteral nutrition; LUQ, left upper quadrant

On POD 13, we performed the daily abdominal ultrasound using the intercostal trans-splenic left upper quadrant (LUQ) view to screen for pleural effusion or ascites. This examination incidentally revealed an oval-shaped, fluid-containing mass (approximately 5 × 10 cm) in the anatomical region where the spleen is normally visualized (Video 1 and Figure 1). The mass exhibited a homogeneous echotexture resembling that of the spleen. However, careful scanning identified the actual spleen separately in a more caudal position. Based on its location and internal characteristics, we interpreted the mass as a distended gastric fundus filled with EN.

**Video 1:** Distended gastric fundus filled with EN Ultrasound video of the gastric fundus obtained from the intercostal trans-splenic LUQ view in the supine position. The gastric fundus is distended and filled with EN EN, enteral nutrition; LUQ, left upper quadrant

Given the persistently high GRV in the preceding days, this sonographic finding was considered to represent the substantial accumulation of gastric contents in the fundus, indicating delayed gastric emptying and an elevated risk of vomiting or aspiration. Based on this assumption, we immediately reduced the continuous EN rate from 30 mL/hour to 5 mL/hour to prevent vomiting and aspiration. We also administered prokinetic therapy with metoclopramide and daikenchuto (a Japanese Kampo herbal formulation) to enhance gastric motility. We did not routinely select postpyloric EN, given ESPEN guideline considerations regarding the technical expertise required for postpyloric tube placement [5].

The same fundus GUS the following day demonstrated the resolution of the distended fundus. EN was subsequently resumed at a low rate under continued ultrasound monitoring, and the target rate was achieved without vomiting and aspiration. Repeated fundus GUS showed no recurrence of gastric retention. The patient remained clinically stable and continued to tolerate EN without complications until POD 22. However, given his underlying lung cancer with empyema, he developed a severe infection and passed away on POD 40.

Sonographic appearance of an EN-filled distended gastric fundus in a healthy volunteer and “pseudo-spleen sign”

**Figure 2:**
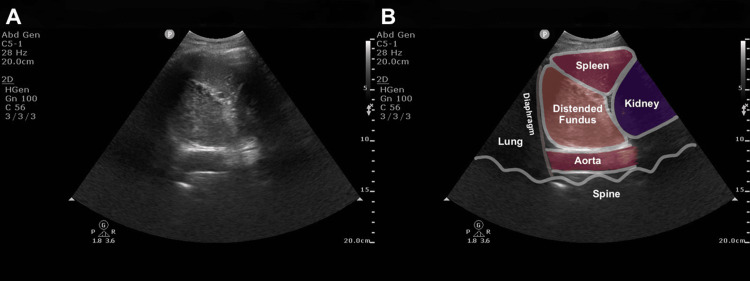
Distended gastric fundus filled with EN in a healthy volunteer Gastric fundus ultrasound image obtained from a healthy volunteer who drank 750 mL of the same EN used for the patient. The image was obtained from the intercostal trans-splenic LUQ view in the supine position. (A) Gastric ultrasound image demonstrating a distended gastric fundus filled with EN. (B) The same ultrasound image with annotation illustrating the distended fundus and surrounding anatomical landmarks EN, enteral nutrition; LUQ, left upper quadrant

In this case, we successfully identified a distended gastric fundus filled with EN using fundus GUS. The enlarged fundus suggested delayed gastric emptying, and its unique sonographic appearance mimicked the spleen. To avoid misidentifying the distended fundus as the spleen, we termed this finding the “pseudo-spleen sign.” To assess the reproducibility of this finding in a pilot manner, we performed fundus GUS in a healthy volunteer who drank 750 mL of the same EN used for this patient and observed the same “pseudo-spleen sign” (Figure 2 and Video 2).

**Video 2:** Distended gastric fundus filled with EN in a healthy volunteer Gastric fundus ultrasound video obtained from a healthy volunteer who drank 750 mL of the same EN used for the patient. The video was obtained from the intercostal trans-splenic LUQ view in the supine position EN, enteral nutrition; LUQ, left upper quadrant

The healthy volunteer was one of the authors (SJ). The EN was a high-protein, peptide-based formula (Peptamen® AF, Nestlé Health Science, Vevey, Switzerland), which is commonly used in critical care settings. Verbal informed consent was obtained from the volunteer (SJ) for participation and for the use of the images and video in this report. The procedure was noninvasive and posed no meaningful risk.

**Figure 3:**
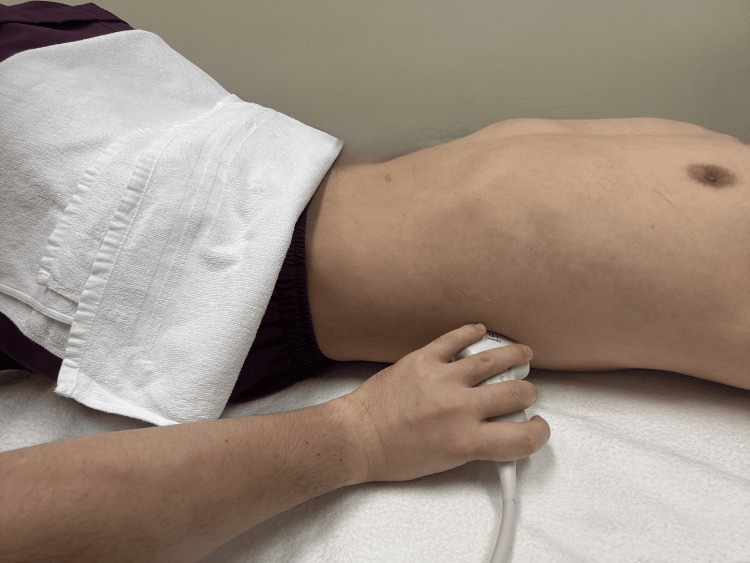
Probe position to scan the gastric fundus using the LUQ view The ultrasound probe is positioned along the ninth or 10th intercostal space on the left mid or posterior axillary line. The anterior or posterior tilting of the probe along the longitudinal axis can be used to detect a distended gastric fundus filled with EN. The photograph was obtained from the healthy volunteer who drank 750 mL of the same EN used for the patient EN, enteral nutrition; LUQ, left upper quadrant

The probe position for gastric fundus scanning using the LUQ view is shown in Figure 3. All GUS examinations were performed by a certified intensive care ultrasonography physician using a portable ultrasound system (CX50, Philips, Amsterdam, Netherlands) with a curved-array transducer or a phased-array transducer.

## Discussion

We identified two important GUS findings in this case.

First, delayed gastric emptying and a subsequent gastric retention can be recognized by visualizing a distended gastric fundus through the LUQ view in the supine position. When supine, the fundus is placed in the most dorsal portion of the stomach. EN retained in the stomach accumulates in the fundus under the influence of gravity, whereas air, which impedes ultrasound visualization, tends to collect in the gastric body and antrum. Given this anatomical configuration, fundal evaluation may serve as a physiologically appropriate alternative to antral assessment when patients cannot be placed in the RLD position. A recent review of gastrointestinal ultrasound published in the Journal of Parenteral and Enteral Nutrition (JPEN) states that GUS should be used to assess gastric emptying and that a standardized protocol for GUS-guided GRV assessment is warranted [1]. The review quoted a previous study that compared GUS-guided GRV monitoring with the standard monitoring of gastric aspirate volume [2]. That study demonstrated that GUS-guided antral assessment significantly reduced the incidence of reflux and aspiration in neurocritical patients. We believe that fundus GUS performed in the supine position may serve as an adjunctive approach for the qualitative assessment of GRV in selected patients when RLD positioning is not feasible.

Second, a distended fundus filled with EN can sonographically resemble the spleen, creating a potential risk of misidentification and misdiagnosis. Unlike clear fluid or blood, an EN formula with a high protein content demonstrates homogeneous echogenicity similar to that of parenchymal organs such as the liver or spleen. When the fundus fills with EN and expands dorsally, it can occupy the space inferior to the left diaphragm, where the spleen is normally located. This creates a spleen-like sonographic appearance, which we term the “pseudo-spleen sign.” We also confirmed the reproducibility of this appearance in a healthy volunteer who drank 750 mL of the same EN in a pilot setting (Figure 2 and Video 2). A previous case report described the successful use of GUS to diagnose upper gastrointestinal bleeding [9]. Using the LUQ view, GUS identified massive intragastric blood accumulation within the fundus, leading to the diagnosis. In that case, the distended fundus contained heterogeneous, not homogenous, contents consisting of blood and clot and therefore was not misinterpreted as a spleen-like mass. In contrast, our case showed a distended fundus filled with homogeneous content, including EN, which closely resembled splenic parenchyma and could potentially lead to misinterpretation. To differentiate an EN-distended fundus from the spleen, color Doppler imaging is useful in confirming the absence of splenic vascular flow within the mass-like structure [10]. Intestinal dilatation related to ileus may also resemble the spleen but can be distinguished by characteristic sonographic features such as plicae circulares of the small intestine and colonic haustra [11,12].

To summarize, while a previous study introduced qualitative fundus GUS as a rapid screening tool to assess gastric content volume before urgent endotracheal intubation in order to exclude a full stomach, our report is the first to highlight the diagnostic value of qualitative fundus GUS for detecting delayed gastric emptying in patients receiving enteral nutrition, with the novel finding of the “pseudo-spleen sign” [8].

There are several limitations in this case report.

First, we observed the “pseudo-spleen sign” in a single patient and one healthy volunteer, and the utility of fundus GUS may be limited in patients with altered gastrointestinal anatomy or large splenomegaly. Therefore, further case series or studies are needed to confirm this sonographic finding and its clinical applicability.

Second, the assessment of the gastric fundus was limited to qualitative evaluation; quantitative approaches may help improve the clinical applicability of this finding.

Third, the success rate of imaging the fundus in the supine position has been reported to be lower than that of antral imaging in the RLD position (44%-67% versus 100% with water intake and 22% versus 28% with solid meal intake, respectively) [7]. Given this relatively low success rate, fundus GUS may serve as an adjunctive approach when antral GUS is inconclusive or not feasible, but it does not replace antral GUS as the primary method for gastric content assessment.

Fourth, we did not perform antral GUS in the semi-recumbent position (head elevated at 45°), which has been reported as an alternative approach [13]. In this case, antral GUS was performed in a supine-based posture with approximately 30° head-up elevation, as recommended for ventilator-associated pneumonia prevention [14]. Because of the patient’s hemodynamic instability, we did not elevate the patient’s position to the 45° semi-recumbent position to perform antral GUS. When patients are hemodynamically stable, antral GUS in the semi-recumbent position should be considered as the first-line alternative approach.

This manuscript adheres to the CAse REport (CARE) guidelines, and the checklist is provided as supplementary material (see Appendices).

## Conclusions

The gastric retention of EN due to delayed gastric emptying can be assessed by fundus GUS using the LUQ view in the supine position. A distended fundus filled with EN can sonographically resemble the spleen, necessitating cautious interpretation to avoid misidentification and misdiagnosis. Fundus GUS in the supine position may serve as a confirmatory or adjunctive approach when antral GUS in the RLD position is inconclusive or not feasible.

## Figures and Tables

**Figure 4:**
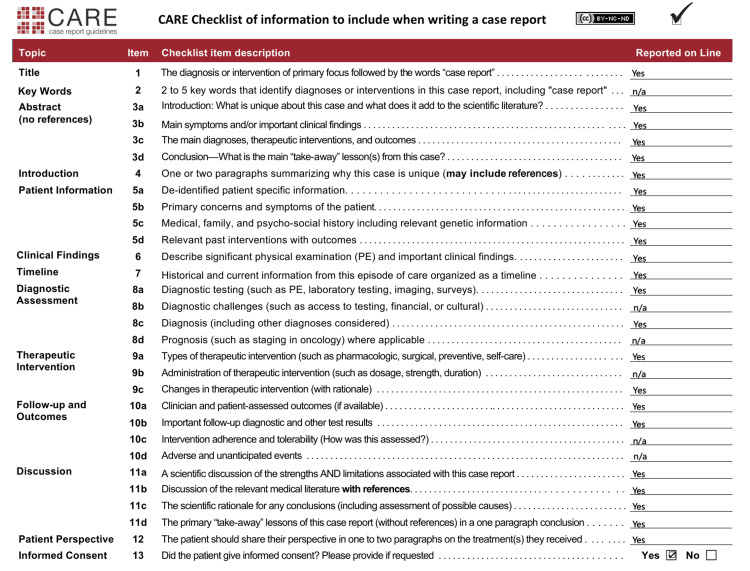
CARE checklist CARE: CAre REport

## Appendices

Figure 4 shows the CARE checklist.
